# Parliament and the Professions

**Published:** 1920-07-10

**Authors:** 


### Parliament and the Professions.

### Tropical Diseases Hospital, Bath.

Mr. Wignall inquired as to the delay in the erection of huts at the Ministry of Pensions Hospital, Bath, for the accommodation of pensioners suffering from tropical diseases, which is stated to cause inconvenience to the medical staff and unnecessary suffering to the men. The First Commissioner of Works explained that the huts were originally required for the accommodation of nurses, who have since been housed elsewhere. It is now proposed, when funds are available, to utilise the huts for additional ward accommodation.

### Nurses in Egypt.

Major Birchall asked the Secretary of State for War whether there are a number of nurses stationed at the 24th Stationary Hospital, Kantara, Egypt, who have been in Egypt for more than three years without leave and whose contracts have expired, and whether arrangements can be made to bring them home? Mr. Churchill said that he had called for a report on the subject.

### Dentistry Bill.

Dr. Addison stated that a Bill is in course of preparation by the Ministry of Health intended to carry out the recommendations of the Departmental Committee on Dentistry which reported in February 1919.

### Part-Time Prison Medical Officers.

The Home Secretary announced that the Treasury have now sanctioned an increase in the remuneration of part-time prison medical officers which, he hoped, will prove satisfactory.

